# Risk of mother-to-child transmission of hepatitis B virus after fetal blood sampling: a report of six cases

**DOI:** 10.1186/s12879-021-06423-x

**Published:** 2021-07-30

**Authors:** Zhenyan Han, Yuan Zhang, Jin Zhou, Qingqing Wang, Yonghua Huang, Hongying Hou

**Affiliations:** 1 Department of Obstetrics and Gynecology, Third Affiliated Hospital of Sun Yat-sen University, No. 600 Tianhe Road, Tianhe District, Guangzhou, 510630 Guangdong Province China; 2 Department of Obstetrics and Gynecology, Jiangmen Central Hospital, Affiliated Jiangmen Hospital of Sun Yat-sen University, Jiangmen, 529030 Guangdong Province China

**Keywords:** Hepatitis B, Mother-to-child transmission, Fetal blood sampling

## Abstract

**Background:**

Hepatitis B virus (HBV) remains a major global public health problem worldwide; in endemic areas, mother-to-child transmission (MTCT) of HBV is the most common transmission route. Previous studies have shown that amniocentesis for prenatal diagnosis increases the risk of MTCT of HBV among highly viraemic mothers. However, no data is available on MTCT related fetal blood sampling (FBS) because of the paucity of cases or lack of attention. We present a case series of HBV-infected women who underwent FBS with or without antiviral therapy during pregnancy and discuss the risk of MTCT after FBS.

**Case presentation:**

Six hepatitis B surface antigen (HBsAg)-positive pregnant women who underwent FBS for prenatal diagnosis were retrospectively reviewed. Their infants were followed up with HBV serology parameters until at least 12 months of age. Among 6 cases, two hepatitis B e-antigen (HBeAg)-positive mothers had high viral loads > 7.0 log_10_ IU/mL, and one of them received antiviral therapy at 26^+ 3^ gestational weeks and achieved an anticipated level of 4.52 log_10_ IU/mL before FBS, while the other one did not receive any antiviral treatment. The other 4 cases were HBeAg-negative with low viral loads. Only a child born to the HBeAg-positive mother, who had no antiviral therapy with a viral load of 7.48 log_10_ IU/mL before FBS, was found to have MTCT with HBsAg persistently positive from birth to 12 months of age. The other 5 children were both HBsAg-negative and HBsAb-positive at the end of follow-up.

**Conclusions:**

FBS may increase the risk of MTCT of HBV in women with HBeAg-positive and high viral loads; therefore, FBS should be avoided in this high-risk population. Maternal HBV serologic testing and awareness of the potential risk of MTCT should be recommended before FBS. Antiviral therapy may be effective to decrease the risk of MTCT after FBS in highly viraemic women.

## Background

Approximately 257 million people worldwide have positive hepatitis B surface antigen (HBsAg), making hepatitis B one of the most widespread chronic infectious diseases and a major global health problem [1]. Mother-to-child transmission (MTCT), either in utero or peripartum, remains the predominant mode of transmission of hepatitis B virus (HBV) from infected mothers to their fetuses or newborns in highly endemic areas. Moreover, the infants who are infected during the first year of life, which is usually caused by MTCT, have a high chronicity rate of approximately 80–90% [1–4]. Thus, preventing MTCT is the most effective way to control the global HBV epidemic.

Since the current hepatitis B immunoglobulin (HBIG) plus HBV vaccine immunisation strategy, which is mainly focused on preventing peripartum MTCT, has been universally implemented in neonates worldwide, the mode of delivery and breastfeeding have been excluded as risk factors for transmission [4–6]. Other concerns on the potential risk of MTCT before birth remain important, for instance invasive prenatal diagnostic procedures [7–9].

Invasive procedures during pregnancy, such as chorionic villus sampling, amniocentesis, and fetal blood sampling (FBS) for prenatal diagnosis, performed before postnatal immunoprophylaxis, can theoretically increase the risk of intrauterine infection of HBV due to placental disruption and maternal blood contamination [7–9]. Recently, the two largest studies, including our matched-cohort data, demonstrated that amniocentesis significantly increased the MTCT rates of HBV in highly viraemic mothers [10, 11]. FBS, still common in developing countries, is mainly performed to access the fetal circulation and obtain samples of fetal blood, usually requiring longer procedure time and introducing more opportunities for maternal blood cells to be transferred to fetal membranes, amniotic cavity, and even circulation [12]. Therefore, FBS may carry a relatively higher risk of MTCT than amniocentesis in women chronically infected with hepatitis B. However, to the best of our knowledge, there are no available reports on MTCT of HBV related to FBS during pregnancy because of the paucity of cases or lack of attention. To address this gap, we reviewed our six HBsAg-positive pregnant women who underwent FBS. Their children were followed up with HBV serology parameters until at least 12 months of age.

## Case presentation

Target cases were derived from pregnant women who were referred to prenatal diagnosis service for various fetal anomalies and had live births in the Third Affiliated Hospital of Sun Yat-sen University and Jiangmen Central Hospital (Jiangmen Hospital of Yat-sen University) between January 2015 and December 2016. Among 298 pregnant women who underwent FBS, six HBsAg-positive mothers and details of their FBS procedures were identified. Maternal and infant data were obtained by reviewing medical records. Six mothers and their infants were followed up in the outpatient service or through telephone interview until at least 12 months after birth (range: 12–38 months), including infant development, immunoprophylaxis, and HBV serological markers. MTCT of HBV was defined by infant HBsAg and/or HBV DNA positivity from birth to the end of the follow-up. This study was approved by the Institutional Review Boards of the Third Affiliated Hospital of Sun Yat-sen University (approval number: [2017] 2–246), and written informed consent was obtained from each participant women.

## CASE 1 with MTCT of HBV after FBS

A 27-year-old primigravida woman was referred to the prenatal diagnosis department for amniocentesis because of a fetal ventricular septal defect (VSD) at 23^+ 3^ gestational weeks in July 2015. The maternal chronic hepatitis B status with HBsAg and HBeAg positivity and an HBV DNA level of 7.48 log_10_ IU/mL were identified. After extensive counselling on the potential risk of MTCT of HBV during the procedure, both parents refused amniocentesis. However, at 26^+ 2^ gestational weeks, the pregnant woman underwent an ultrasound guided fixed-needle FBS with placental penetration from a free loop in another tertiary center. Transient post-procedure bleeding from the punctured umbilical cord and placenta was recorded. The results of single nucleotide polymorphism (SNP) array and karyotypes were normal. At 39^+ 5^ weeks of gestation, a male neonate was delivered vaginally with HBsAg and HBeAg positive, and a small VSD without other cardiac abnormality was confirmed by postnatal echocardiography. The infant received 200 IU of HBIG and 10 μg of yeast-derived recombinant HBV vaccine within 6 h after birth, and additional doses of HBV vaccine were administered at ages 1 and 6 months. In the follow-up examination at the age of 12 months, serum HBsAg was persistently positive (Table 1), while serum alanine aminotransferase levels were normal.

**Table 1:** Characteristics of HBsAg-positive pregnant women who underwent fetal blood sampling and outcomes of infants

Characteristics and Outcomes	Case series					
	CASE 1	CASE 2	CASE 3	CASE 4	CASE 5	CASE 6
Mothers						
Maternal age, years	27	26	30	34	35	28
Gravida/Parity	1/1	1/1	1/1	5/2	3/3	1/1
Antiviral therapy	No	Yes^a^	No	No	No	No
HBeAg status	Positive	Positive	Negative	Negative	Negative	Negative
Baseline HBV DNA levels, log_10_ IU/ml	7.48	4.52	Undetected	Undetected	2.51	2.52
Mode of delivery	Vaginal delivery	Vaginal delivery	Vaginal delivery	Caesarean section	Vaginal delivery	Vaginal delivery
Gestational weeks at delivery	39^+ 5^	41	39^+ 4^	39^+ 2^	32^+ 1^	38^+ 3^
Obstetric complications	No	No	No	No	Preterm birth	No
FBS Technique						
Gestational weeks	26^+ 2^	32^+ 4^	31^+ 3^	32^+ 4^	29^+ 2^	25^+ 3^
Indications	Ventricular septal defect	Ventriculomegaly	Suboccipital cystic mass	Fetal growth restriction	Hydronephrosis	Ventricular septal defect
Transplacental penetration	Yes	No	No	No	No	No
Post-procedure bleeding (from placenta or uterine wall)	Yes	No	No	No	No	No
Post-procedure streaming (from punctured umbilical cord)	Yes	Yes	Yes	Yes	Yes	Yes
Infants						
Sex	Male	Male	Male	Male	Female	Female
Birthweight, gram	3100	2950	3200	2600	1900	3400
HBsAg status at birth	Positive	Negative	Negative	Negative	Negative	Negative
Timing of Follow-up, months	12	12	29	32	12	38
HBsAg status at the end of follow-up	Positive	Negative	Negative	Negative	Negative	Negative
Anti-HBs status at the end of follow-up	Negative	Positive	Positive	Positive	Positive	Positive

^a^Tenofovir disoproxil fumarate 300 mg daily from 26^+ 3^ weeks of gestation to postpartum week 4

## CASES 2–6 with no MTCT of HBV after FBS

The other 5 HBV-infected pregnant women underwent FBS at 25^+ 3^–32^+ 4^ gestational weeks because of various fetal anomalies, including ventriculomegaly, suboccipital cystic mass, fetal growth restriction (FGR), hydronephrosis and VSD. Case 2 was a chronic hepatitis B patient with immune tolerant phase for 8 years before pregnancy. At the first trimester, the examination confirmed HBeAg positivity and HBV DNA of 7.56 log_10_ IU/mL. The antiviral therapy with tenofovir disoproxil fumarate (TDF) 300 mg daily was initiated at 26^+ 3^ weeks of gestation to postpartum week 4, and the viral load was decreased to 4.52 log_10_ IU/mL before FBS. The other 4 cases were all HBsAg-positive and HBeAg-negative. After the counselling and consent, ultrasound-guided freehand techniques were conducted in all 5 cases uneventfully without placental penetration and post-procedure bleeding, and all the results of SNP-array and karyotype were normal. One pregnant woman went into spontaneous preterm labor at 32^+ 1^ gestational weeks and had a normal vaginal delivery of a female baby with a birth weight of 1900 g. The other 4 mothers experienced uneventful obstetric conditions and term delivery. All 5 infants received a timely birth dose of HBIG and HBV vaccine and standard primary immunisation of HBV afterward. On follow-up, serum HBsAg was persistently negative in all the 5 infants from birth to 12–38 months (Table 1).

## Discussion and conclusions

Although the number of FBS has greatly decreased because of the emergence of earlier, more accurate, and safer diagnostic modalities [12], in some centers with a high prevalence of certain genetic diseases, limited molecular diagnostic techniques, and the very delayed abnormal sonographic findings, FBS could not be completely replaced by chorionic villus sampling or amniocentesis [13, 14]. Most reported studies on FBS mainly focused on the procedure-related safety and efficacy [13–16]; however, the FBS-related risk of MTCT of blood-borne viruses, such as HBV, has not been assessed [7]. The present case series with one HBV-infected infant born to a HBeAg-positive and highly viraemic mother highlights the non-negligible concern on this issue.

HBV is the mostly transmitted from infected mothers to children during childbirth through contact with blood and other body fluids [1]. After implementation of universal HBIG plus HBV vaccine strategy, which is mainly targeted to the peripartum MTCT, intrauterine infection become an important cause of postnatal immunoprophylaxis failure. The possible mechanisms of the intrauterine infection include transplacental leakage, placental infection, peripheral blood leukocyte infection, and germline cell infection [17]. Invasive diagnostic procedures during pregnancy may imply the potential risk of transmission of HBV because of disruption of the maternal-fetal surface through needle passage and leading to placental leakage. The findings of two recent studies on the risk of MTCT after amniocentesis confirmed this hypothesis [10, 11]. FBS is a relatively invasive and time-consuming technique due to its penetration through one or more tissue layers into the umbilical cord than amniocentesis. Therefore, it theoretically has more potential for fetal exposure to maternal blood cells and higher risk of MTCT of HBV. In this case series, one in six infants born to an untreated HBsAg-positive mother who underwent transplacental FBS with post-procedure bleeding was finally considered as the MTCT case. This indicated that FBS might increase the risk of MTCT of HBV, and transplacental procedures should be avoided.

Similarly, the risk factors of MTCT after amniocentesis included maternal HBeAg-positive and HBV DNA > 7 log_10_ IU/mL [10, 11], which were also the most important features of this MTCT case after FBS. Serum HBV DNA level and HBeAg status are the two best indicators of viral replication; thus, high maternal viraemia and HBeAg positivity have always been the most prominent predictors for MTCT [2–4, 18, 19]. Antiviral agents, such as TDF, telbivudine, and lamivudine, can efficiently inhibit maternal HBV replication and significantly reduce MTCT rates [18, 20]. Moreover, no MTCT events occurred among women with HBeAg-negative or HBV DNA < 7 log_10_ IU/mL after invasive procedures in our previous study [11] and current case series. Presumably, short-term antiviral therapy to obtain the optimal threshold of HBV DNA levels before the procedure may be feasible to prevent MTCT. However, only one available study reported on this preventive strategy, without reaching statistical significance, a trend of decreasing MTCT rates was observed when amniocentesis was performed in highly viraemic mothers with antiviral therapy [11]. Meanwhile, in our case series, one HBeAg-positive mother with HBV DNA of 7.56 log_10_ IU/mL, after 6 weeks TDF treatment, achieved a relative low level of 4.52 log_10_ IU/mL before FBS, and her infant was persistent HBsAg-negative from birth to the age of 12 months. As for optimal maternal target viral load before invasive procedure, especially FBS, there is currently no recommendation and guideline due to paucity of data. In fact, it is controversial to use the recommended DNA level of 5.3 log_10_ IU/mL (200,000 IU/mL) before delivery [18] as an anticipated level before invasive procedures to prevent MTCT, because the amniocentesis and FBS are usually performed at 15–24 weeks and more than 20 weeks, respectively, which is well before the timing for postnatal immunoprophylaxis, even the risk of contact maternal blood is lower than in childbirth. Meanwhile, initiation of antiviral therapy in early pregnancy is another concern. To prevent MTCT among highly viraemic mothers, most international guidelines recommend the TDF treatment start at 24–28 weeks of gestation [18]. However, antiviral therapy before FBS may be initiated earlier than 24 gestational weeks. The safety issues associated with a relative longer fetal exposure to TDF including renal and bone toxicity should be cautious, even most studies on fetal TDF exposure found no significant risk of the adverse effects during childhood [21]. Therefore, the use of maternal antiviral treatment before FBS to prevent MTCT should be further studied in details.

On the other hand, it is worth noting that, with a higher HBsAg prevalence of 5–7.99%, both Thailand and China had been ranked as the high-intermediate endemic regions [22], where hundreds and thousands of FBS have been performed in many referral centers [12–16] although most data have not been published. MTCT risk assessment after FBS and feasible preventive strategies may be impossible if professionals are unaware of maternal HBV infection or MTCT risks. Therefore, maternal HBV serologic testing and awareness of the potential risk of MTCT before invasive procedures, including amniocentesis and FBS, should be the first step to consult patients and prevent transmission.

In summary, although no accurate rate of FBS-related MTCT of HBV can be drawn from these limited data, this case series highlights the concern of this issue and demonstrates that FBS may increase the risk of MTCT of HBV in women with HBeAg-positive and high viral loads. FBS, especially transplacental techniques, should be avoided in this high-risk population. Non-invasive cell-free DNA screening may be an alternative to these women but limitation of this screening should be fully informed and counselled. Meanwhile, antiviral therapy may be effective to decrease the risk of MTCT after FBS in highly viraemic women. Further studies that include more FBS cases with hepatitis B are needed to characterize the risk of MTCT after FBS.

## Data Availability

The datasets used and/or analysed during the current study are available from the corresponding author on reasonable request.

## Abbreviations

HBsAg: hepatitis B surface antigen
MTCT: mother-to-child transmission
FBS: fetal blood sampling
HBeAg: hepatitis B e-antigen
HBIG: hepatitis B immunoglobulin
VSD: ventricular septal defect
SNP: single nucleotide polymorphism
FGR: fetal growth restriction
TDF: tenofovir disoproxil fumarate
